# Quantifying Regional Differences in the Length of Twitter Messages

**DOI:** 10.1371/journal.pone.0122278

**Published:** 2015-04-08

**Authors:** Christian M. Alis, May T. Lim, Helen Susannah Moat, Daniele Barchiesi, Tobias Preis, Steven R. Bishop

**Affiliations:** 1 Department of Mathematics, University College London, London, United Kingdom; 2 National Institute of Physics, University of the Philippines Diliman, Quezon City, Philippines; 3 Warwick Business School, University of Warwick, Coventry, United Kingdom; UNMdP-CONICET, ARGENTINA

## Abstract

The increasing usage of social media for conversations, together with the availability of its data to researchers, provides an opportunity to study human conversations on a large scale. *Twitter*, which allows its users to post messages of up to a limit of 140 characters, is one such social media. Previous studies of utterances in books, movies and *Twitter* have shown that most of these utterances, when transcribed, are much shorter than 140 characters. Furthermore, the median length of *Twitter* messages was found to vary across US states. Here, we investigate whether the length of *Twitter* messages varies across different regions in the UK. We find that the median message length, depending on grouping, can differ by up to 2 characters.

## Introduction

As more people turn online to communicate or to seek information, the possibility of understanding their offline behaviour by means of their online digital traces becomes more appealing. Previous studies employing these digital traces allowed researchers to test hypotheses on happiness [1], social influence [2, 3] and social organization [4], gain insights on decision making in stock markets [5–7] and elections [8], and quantify social phenomena [9]. Online social media and search engine query data not only allow researchers to detect events happening in the present [10–13], but also enable them to make predictions about the future [14, 15]. The ubiquity of social media has been useful in investigating disasters [16, 17], which may help in saving human lives (see [18] for a review). Indeed, datasets generated from online activities of people are important resources in the field of computational social science [19, 20]. Even the digitisation of large amounts of offline information is also useful as it has enabled researchers to study language [21] and scientific progress [22, 23],

*Twitter* is a social media platform that allows its users to post messages (*tweets*) of up to 140 characters, which are public by default. It is one of the most popular online social media with 255 million average monthly users as of 31 March 2014 [24]. According to the Ipsos MediaCT Tech Tracker report [25], 18% of adults in the UK visited Twitter in the third quarter of 2014. Owing to its popularity and availability of data, *Twitter* is being used as a tool to study social systems [1, 4, 26–31] and language [32–35].

The 140-character length limit of tweets does not seem to affect the length of most conversation messages on *Twitter*. In *Twitter*, the median conversational message length is 38 characters while in books it is 48 characters, and 25 characters in movies [36]. If the same length limit of 140 characters was imposed on books and movies, then only 8.96% of the messages in the former and 0.012% of messages in the latter would reach the limit.

Public conversations on *Twitter* are typically performed using replies, which are tweets that start with the usernames of the recipients. An analysis of the content of tweets posted in the US [37] showed regional variations in slang while an analysis of replies posted in the US [38] found correlation of message lengths to a particular demographic variable instead of location.

Several studies in England [39–41] have shown economic and health differences between the north and south. There are critics, however, opposing the idea of the existence of a North-South divide because boundaries of different language features do not coincide [42] or the boundary changes depending on the political motives of the one assigning it [43].

In England there is also a common stereotype that people in the North talk more than those in the South. The perception is that people living north of some, possibly indeterminate, line that separates north from south, are friendlier than their southern counterparts and hence end up talking more. Motivated by the availability of data and the possibility of observing a North-South divide which provides evidence of the stereotype around chattiness, we looked at how the lengths of the messages in conversational tweets (replies) differ across various geographical groupings. We were able to consider 3,443,773 messages posted throughout the various regions of the UK. However, we found no significant evidence of a North-South divide in the message lengths.

## Results

### Message length in terms of absolute length

**Fig 1:**
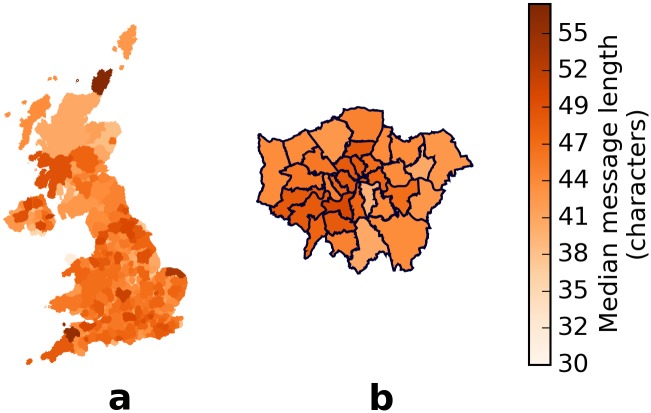
Message lengths, in characters, of different districts in UK. Although the median message lengths ranged from 30 to 57.5 characters, 90% are within 11 characters and 50% are within 4 characters. This homogeneity in the median message length is observed when plotted on a map of the (a) entire United Kingdom and of the (b) boroughs of Greater London. There is also no apparent grouping of districts, by latitude or otherwise.

Although the median message length of conversational tweets (replies) from administrative districts in the UK ranged from 30 to 57.5 characters, 90% of the districts have median message lengths between 39 to 50 characters, and 50% are between 43 to 47 characters. Visualisation of the median message length across administrative districts in the UK does not show any obvious grouping of districts (Fig. 1a) even in Greater London (Fig. 1b). As previously observed [36, 38], the message length distributions are skewed (S1 Fig.). At least 75% of the messages in each district have a message length of at most 90 characters, which is 64% of the length limit, or 73% of the available limit after subtracting the 15-character limit of a *Twitter* username and one @ sign.

**Fig 2:**
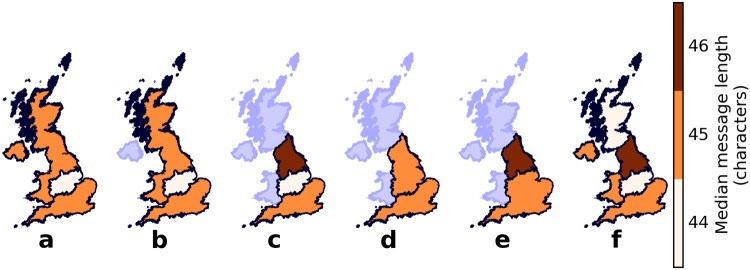
Median message lengths, in characters, for different groupings of districts. Combining Northern England with other parts of UK to form (a) Northern UK and (b) Northern Great Britain results in median message lengths equal to that of Southern England. (c) Northern England, by itself, would have the longest median message length but (d) grouping it with the Midlands results in the same median message length as for Southern England. The median message length of the (e) union of the Midlands and Southern England is smaller by one character than that of Northern England, consistent with the stereotype. Computing the median message lengths of the (f) other home countries yields three groups: Southern England, Wales and Northern Ireland, the Midlands and Scotland, and Northern England.

Grouping the tweets into Southern England, the Midlands and Northern UK (that is, Northern England, Scotland and Northern Ireland) reveals a 1-character difference in the median message length (Fig. 2a) of the Midlands (44 characters) compared to the rest of UK, which is very small but statistically significant (Kruskal-Wallis, *H* = 471.2, *p* < 0.001, *n* = 3, *N* = 3,443,773). Note that for all statistics reported throughout this manuscript, *p* refers to the *p*-value associated with the reported statistic. We also investigate the properties of a Northern Great Britain group, created by excluding Northern Ireland from the Northern UK group. However, this results in the same median message length (Fig. 2b) as for Northern UK. Further excluding Scotland and Wales from the Northern Great Britain group yields a median message length (Fig. 2c) of 46 characters, which is greater than both the Midlands and Southern England, and almost consistent with the stereotype.

Combining the Midlands with Northern England as in Ref. [41], results in both Northern and Southern England groups (Fig. 2d) having the same median message length of 45 characters. On the other hand, combining the Midlands with Southern England (Fig. 2e) results in the median message length for Northern England being larger by one character, consistent with the stereotype. However, we note that this difference is extremely small.

These results suggest that the grouping is perhaps not between North and South but something else. With that in mind, we repeat the analysis but this time treat Wales, Scotland and Northern Ireland as separate from Northern England, the Midlands and Southern England. The median message lengths of this new grouping are shown in Fig. 2f, which implies three groups: Southern England, Wales and Northern Ireland, the Midlands and Scotland, and Northern Ireland.

**Fig 3:**
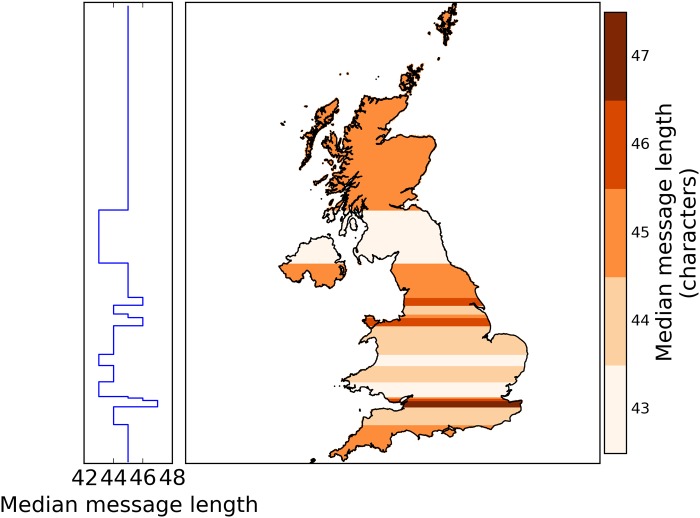
Median message length, in characters, by latitude. Partitioning the tweets into 20 latitude bins of about 10^5^ tweets and 10^4^ users each does not show any North-South division, however, the similarity of the Midlands and Southern Scotland becomes more prominent. Changing the number of bins does not result in drastic qualitative changes.

Grouping the tweets by latitude (Fig. 3) further emphasises that those posted from the Midlands are shorter than those from the rest of UK. The characteristic shorter tweets from Scotland, which made it similar to the Midlands, turn out to be valid only for Southern Scotland.

The distribution of the number of tweets per user in the dataset (S2 Fig.) is very skewed with 39% of users having only one tweet in the dataset whilst one particular user has 4,178 tweets. To check the robustness of the results, we impose minimum and maximum thresholds per user and then analyse only tweets from users who passed the thresholds. The results of applying a range of thresholds are tabulated in Table 1. It is worth noting that the threshold of at least 20 tweets per user was imposed in the study on geographical lexical variation by Eisenstein et al. [37].

**Table 1:** Median message length, in characters, for different geographical regions after imposing user tweet count thresholds.

Region	Allowed number of tweets per user						
	> 0	5–10	≥ 5	5–20	≥ 10	10–20	≥ 20
Southern England	45	47	45	46	45	46	45
The Midlands	44	45	44	45	44	45	43
Northern UK	45	46	45	46	45	46	45
Northern GB	45	46	45	46	45	46	45
Northern England	46	47	46	47	46	47	45
Wales	45	46	45	46	44	46	44
Scotland	44	45	44	45	44	45	44
Northern Ireland	45	47	45	47	45	47	45
Total tweets	3,443,773	374,202	2,988,695	742,393	2,664,913	418,611	2,274,762

The median message lengths do not remain constant when we impose these thresholds, but varies by as much as 2 characters for most of the imposed thresholds. Nevertheless, the main observations that Southern and Northern England, and the Midlands and Scotland, have the same or almost the same median message lengths remains.

The use of characters as unit of message length is especially suitable for tweets because there is a 140-character limit. It is also more sensitive to differences in orthography, making it easier to detect differences in language. Indeed, using the number of words as unit would result to almost all groups having a median message length of 9 words (Table 2). Only the Midlands has some variation, having a median message length of 8 words for three tweet count thresholds (≥ 5, ≥ 10 and ≥ 20 tweets per user). In all pairs of user count thresholds, the median message lengths of Southern England and Northern England are the same.

**Table 2:** Median message length, in words, for different geographical regions after imposing user tweet count thresholds.

Region	Allowed number of tweets per user						
	> 0	5–10	≥ 5	5–20	≥ 10	10–20	≥ 20
Southern England	9	9	9	9	9	9	9
The Midlands	9	9	8	9	8	9	8
Northern UK	9	9	9	9	9	9	9
Northern GB	9	9	9	9	9	9	9
Northern England	9	9	9	9	9	9	9
Wales	9	9	9	9	9	9	9
Scotland	9	9	9	9	9	9	9
Northern Ireland	9	9	9	9	9	9	9

### Message length in terms of available space

Although a tweet can be up to 140 characters long, here we only consider replies. The maximum length of a message is therefore smaller than 140 characters because the recipient usernames which begin a reply take up some of the available space. A user may then be forced to shorten their message because of the reduced space.

**Fig 4:**
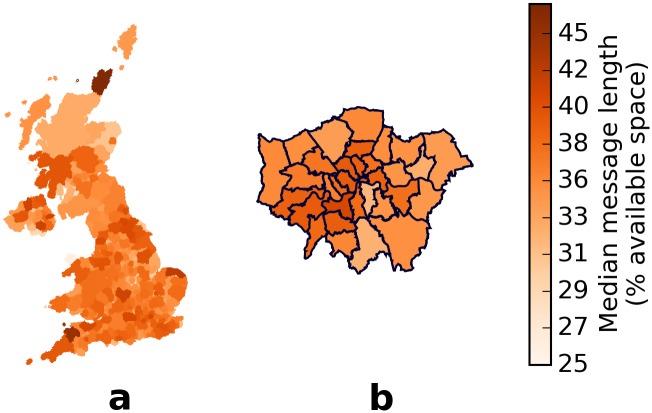
Message lengths, in terms of available space, of different districts in UK. The median message length, in terms of available space, of each administrative district in the (a) entire United Kingdom hardly varies even across the (b) boroughs of Greater London. The median message lengths of each district measured by characters and by available space are almost perfectly correlated (Spearman *ρ* = 0.99, *n* = 404, *p* < 0.001).

**Fig 5:**
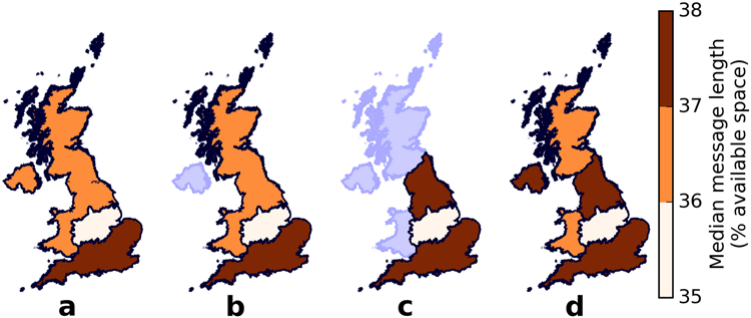
Median message lengths, in terms of available space, for different groupings of districts. Combining Northern England with other parts of UK to form (a) Northern UK and (b) Northern Great Britain results in median message lengths equal to that of Southern England. (c) Northern England, by itself, also has the same median message length as for Southern England. Computing the median message lengths of the (d) other home countries and doing pairwise Kolmogorov-Smirnov tests with Bonferroni correction (*α* = 0.05, *n* = 15) yields three clusters: the Midlands and Scotland, Wales, and rest of UK.

**Fig 6:**
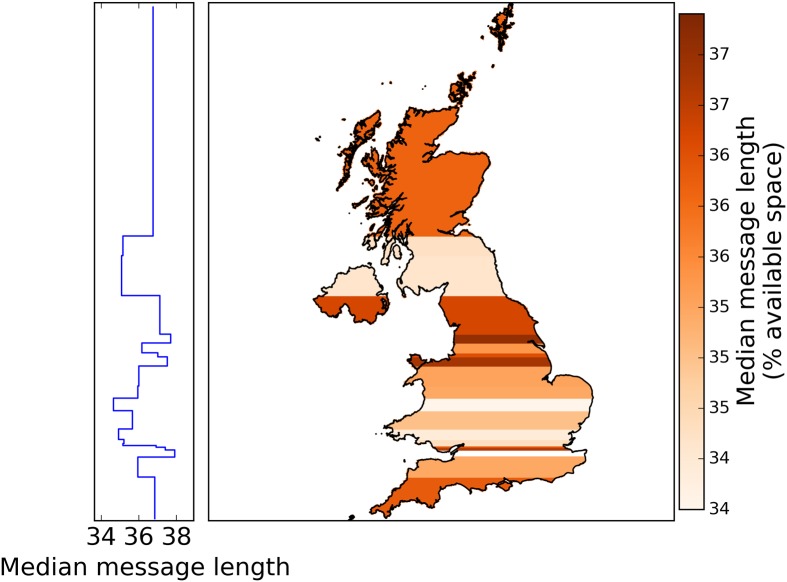
Median message length, in characters, by latitude. Partitioning the tweets into 20 latitude bins of about 10^5^ tweets as in Fig. 3 similarly did not show any North-South division. The median message length per latitude bin measured by characters and by available space are also almost perfectly correlated (Spearman *ρ* = 0.97, *n* = 20, *p* < 0.001).

When measuring the message lengths in terms of the available space, we do not find any obvious North-South division in the districts of the entire UK (Fig. 4a) or even in the boroughs of Greater London (Fig. 4b). The median message lengths of each district measured by characters and by available space are almost perfectly correlated (Spearman *ρ* = 0.99, *n* = 404, *p* < 0.001). Both Northern UK (Fig. 5a) and Northern Great Britain (Fig. 5b) have smaller median message lengths than that of Southern England but longer than that of the Midlands. Unlike in Fig. 2c where the median message length of Northern England is longer than that of Southern England, in Fig. 5c the median message length of Northern England is the same as that of Southern England. Unlike before, the median message length (Fig. 5d) of Scotland is within the same range (36–37% available space) of Wales instead of the Midlands. Binning the tweets into latitudes (Fig. 6) does not reveal any qualitative difference from Fig. 3. The median message length per latitude bin measured by characters and by available space are almost perfectly correlated (Spearman *ρ* = 0.97, *n* = 20, *p* < 0.001).

The median message lengths in terms of available space are always below 50%. That is, there is ample space for messages despite being shortened by leading usernames in the tweet. The percentages of messages that used up at least 90% of the available space is only 10%. Considering that the median message lengths of conversations in unconstrained media are 48 characters for books and 25 characters for movies, and that 8.96% and 0.012% of the conversational messages in those media, respectively, exceed 140 characters, the low utilisation of available space is not surprising. The length limit of tweets is simply enough for most tweets.

## Discussion

We did not find evidence of a difference in the length of tweets between the North and South of the UK within our sample of Twitter messages. At best, the divide is not between North and South, but the Midlands and Scotland, and the rest of UK. The difference we found in these cases, however, is only 1 or 2 characters.

## Materials and Methods

Using the *Twitter* application programming interface [44], we retrieved, depending on the date, 1% to 15% of public tweets from 19 November 2009, the first day that tweet coordinates were provided, to 20 December 2012. Due to data collection issues, there were missing days in the collection period but only days that were complete were considered in the data analysis, resulting in 839 days analysed. The dataset was then filtered for conversational tweets (replies) posted from the United Kingdom. The list of tweet IDs analysed in this paper was deposited on figshare (http://dx.doi.org/10.6084/m9.figshare.1249692)

The message of each filtered tweet was extracted by removing all leading @usernames, and leading and trailing whitespaces of the remaining text. Leading whitespaces were not removed when the message length was measured in terms of the available space because some tweets do not use whitespaces to separate the @usernames with the message. Messages with a length, in characters, of zero or greater than 140 (maximum allowed length of tweets) were then discarded resulting in a total of 3,443,773 tweets posted by 372,783 users, suitable for analysis by region. The length of messages in words was determined by calculating the number of chunks after splitting the message by whitespaces.

The posting location of a tweet was determined by using its geo, coordinates and place metadata (geotags), where these fields were considered in this order. Tweets were then assigned to individual districts based on boundary data provided by the UK Ordnance Survey.

Tweets that only have place information were considered to be from UK if the country attribute of place was United Kingdom. The centroid of the bounding_box attribute, if it existed, were then used to determine the districts. Otherwise, the tweet was assigned to the name of the place if the place_type was city or admin. A tweet with no coordinates and only England as its place was excluded because the location information was too coarse to determine if it came from Northern England, Southern England or the Midlands. Note that *Twitter* users must opt-in to have their location information included in their tweet metadata.

## Supporting Information

S1 Fig Message length distribution for each district in the United Kingdom, arranged by increasing median message length. The dark blue center line indicates the median while the lighter blue region is bounded by the 25th to 75th percentiles. The lightest blue region is bounded by the extrema. At least 75% of the messages in each district have a message length of at most 90 characters, which is 64% of the length limit, or 73% of the available limit after subtracting the 15-character limit of a *Twitter* username and one @ sign.
(TIF)

S2 Fig Number of tweets per user in the dataset. As expected, both (a) histogram and (b) complementary cumulative distribution exhibit a skewness in the distribution of number of tweets per user.
(TIF)
