# Supplementary figures and images for: Quantifying Regional Differences in the Length of Twitter Messages
Source: PLoS One. 2015 Apr 8;10(4):e0122278. doi: 10.1371/journal.pone.0122278 (PMC4390308; doi:10.1371/journal.pone.0122278)

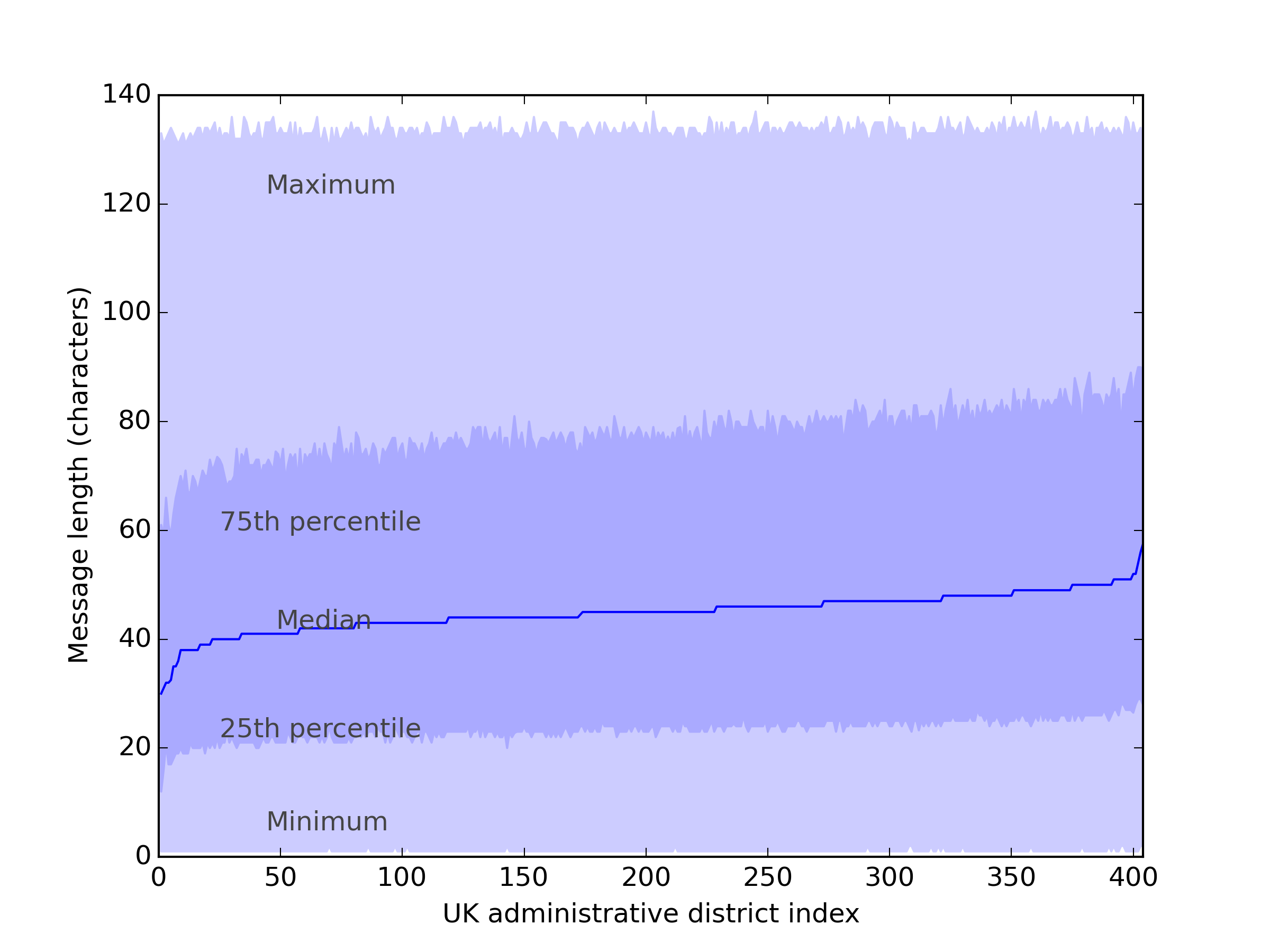

Supplement: S1 Fig — The dark blue center line indicates the median while the lighter blue region is bounded by the 25th to 75th percentiles. The lightest blue region is bounded by the extrema. At least 75% of the messages in each district have a message length of at most 90 characters, which is 64% of the length limit, or 73% of the available limit after subtracting the 15-character limit of a Twitter username and one @ sign. (TIF) [file pone.0122278.s001.tif]

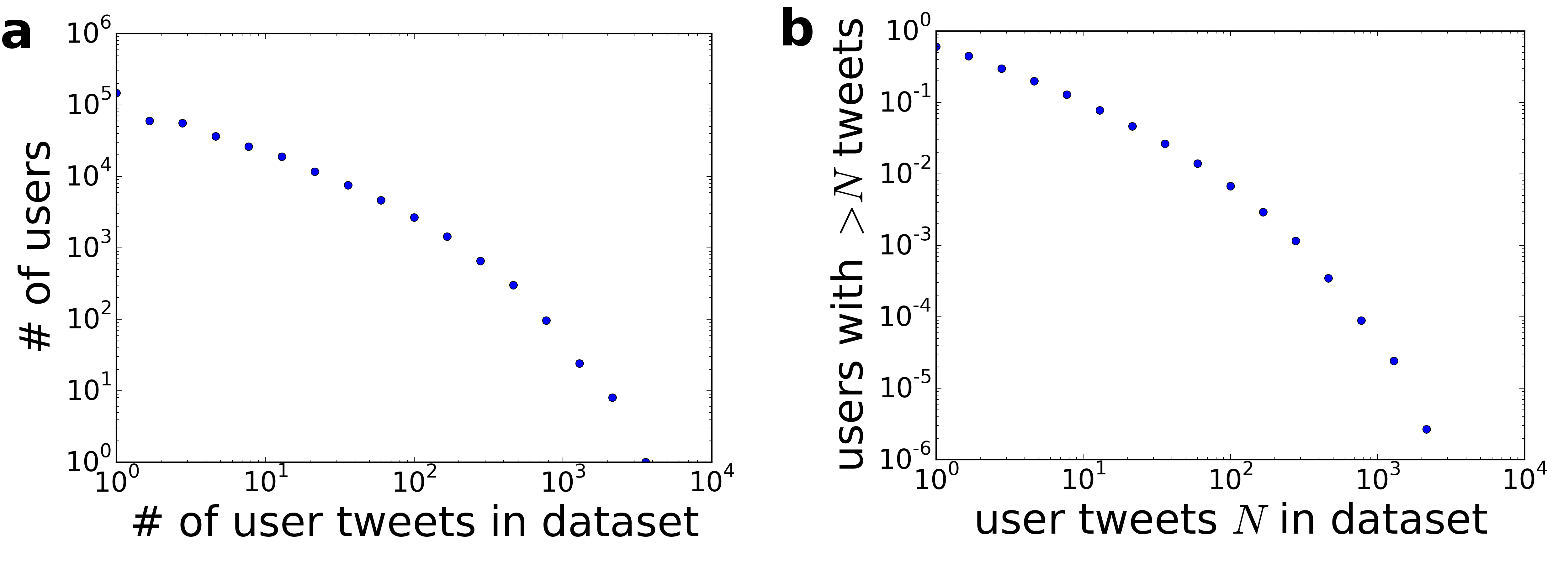

Supplement: S2 Fig — As expected, both (a) histogram and (b) complementary cumulative distribution exhibit a skewness in the distribution of number of tweets per user. (TIF) [file pone.0122278.s002.tif]
